# Vanishing fine structure splitting in highly asymmetric InAs/InP quantum dots without wetting layer

**DOI:** 10.1038/s41598-020-70156-1

**Published:** 2020-08-11

**Authors:** Michał Zieliński

**Affiliations:** Institute of Physics, Faculty of Physics, Astronomy and Informatics, Nicolaus Copernicus University, ul. Grudziadzka 5, 87-100 Toruń, Poland

**Keywords:** Atomistic models, Quantum dots

## Abstract

Contrary to simplified theoretical models, atomistic calculations presented here reveal that sufficiently large in-plane shape elongation of quantum dots can not only decrease, but even reverse the splitting of the two lowest optically active excitonic states. Such a surprising cancellation of bright-exciton splitting occurs for shape-anisotropic nanostructures with realistic elongation ratios, yet without a wetting layer, which plays here a vital role. However, this non-trivial effect due to shape-elongation is strongly diminished by alloy randomness resulting from intermixing of InAs quantum-dot material with the surrounding InP matrix. Alloying randomizes, and to some degree flattens the shape dependence of fine-structure splitting giving a practical justification for the application of simplified theories. Finally, we find that the dark-exciton spectra are rather weakly affected by alloying and are dominated by the effects of lateral elongation.

## Introduction

Quantum dots^1,2^ are man-made semiconductor nanostructures that come in a wide variety of types^3–5^, and are extensively studied with interest driven by both basic scientific curiosity as well as promising applications in quantum information^6^, computing^7,8^, and cryptography^9^. Apart from the elementary excitations, electrons and holes, quantum dots can confine interacting electron-hole pairs, namely excitons.^10^ An emission cascade from a two exciton (biexciton) state, through two indistinguishable exciton states should lead to the emission of polarization entangled photon pairs.^9,11–13^ However, in realistic quantum dots the intermediate exciton state is often split by the electron-hole exchange interaction^14–16^ hindering the efficiency of the entanglement generation. This energetic difference between the two bright exciton states, known as the fine-structure splitting or the bright exciton splitting (BES) is typically (10–$100\,{\upmu }\hbox {eV}$) much larger than the emission linewidth ($\sim 1\,{\upmu }\hbox {eV}$). Tailoring the BES in nanostructures is thus essential due to its relevance for photon entanglement generation.^7,11,17,18^ This is particularly important for InAs/InP nanostructures, which are promising candidates for quantum emitters at 1.3 or $1.55\,{\upmu }\hbox {m}$ telecommunication relevant wavelengths^19–22^. Notably, InAs/InP nanostructures can be fabricated in various ways, including self-assembled and nanowire quantum dots^23–27^ of quasi-cylindrical shapes, as well as strongly elongated dots^28–42^, sometimes referred to as quantum dashes. Similarly to cylindrically-shaped dot systems, quantum dashes have the potential for applications^30,31,43–45^ combined with considerable tuning capabilities^46,47^. The manipulation of the BES aimed towards generation of entangled photon pairs is a vital and broad research area with significant efforts have been made utilizing post-growth annealing^48,49^, spectral filtering^9^, sample selection^50,51^, growth of highly symmetric structures^24,26,52–54^, and the application of external electric^55,56^, magnetic^18,57,58^, and strain fields^59–61^. With respect to the above quantum dashes are rather unobvious candidates for entanglement generation, since the BES is these systems is expected to be rather substantial^47,62,63^ due to their pronounced shape elongation^16,64^. However, in this work we show that shape elongation may not only reduce the bright exciton splitting^63^, but even lead to its vanishing, and subsequently to the reversal of bright excitonic spectral lines. This is possible for large but realistic elongation ratios, and thus practical quantum dash dimensions, yet for the growth process that does not incorporate the wetting layer. Apart from approaches based on droplet epitaxy^65,66^, obtaining such nanostructures seems at first very unlikely in the Stransky-Krastanov growth mode. However, recently an effective decoupling of the wetting layer from the quantum dot has been achieved by the addition of a monolayer of AlAs following the InAs quantum dot formation^67^. Ref.^67^ thus opens possibilities of obtaining Stransky-Krastanov assembled nanostructures with highly limited role of the wetting layer. Following these recent experimental efforts, we exploit such concept theoretically in this paper, by considering elongated quantum dot systems, yet grown without the wetting layer, conceivably with prospects of experimental realization of such quantum dots-quantum dash structures in near future.

No self-assembled quantum dash (or quantum dot) can be grown without the mixing of nanostructure material with the material of the surrounding matrix. This composition mixing, or alloying, leads to related alloy randomness, which usually affects the bright exciton spectra in a profound way^68,69^. In this work, we show that for a relatively small ($P=10\%$ or 20%) amount of phosphorous migrating into the InAs quantum dash from the surrounding InP matrix is it still possible to observe the reduction of the BES for elongation aspect ratio values between 6 and 8. However, for a larger degree of alloying, $P=50\%$, random fluctuations seem to dominate and cancel the discussed effect.

Finally, we study dark-exciton spectra^15,70–72^, properties of which in quantum dashes, such as the non-zero optical activity^63,73^ and splitting, appear to be dominated by shape deformation and rather immune to alloying effects.

## Results

**Figure 1:**
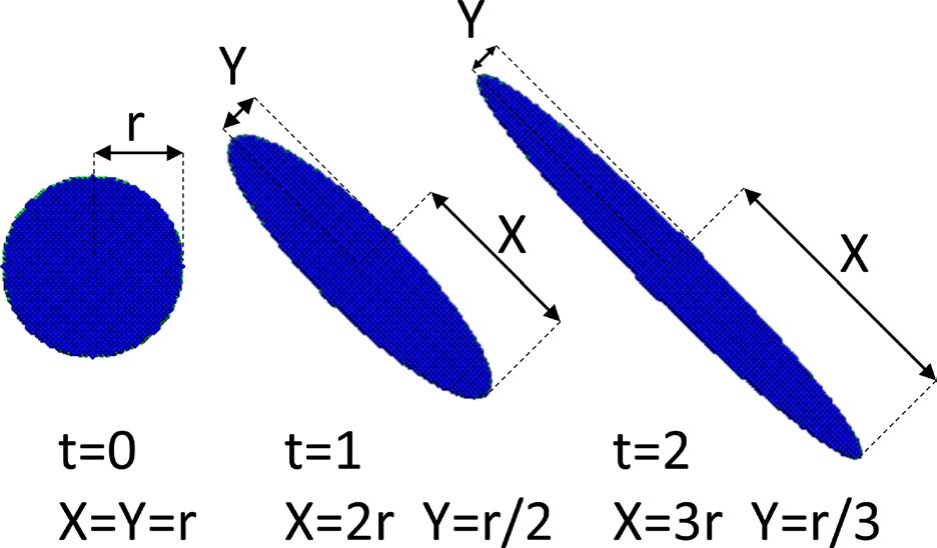
Schematics: top-view of an InAs nanostructure with shape deformation along the [$1{\bar{1}}0$] crystal axis as a function *t* for $t=0$, 1 and 2. The surrounding InP barrier is invisible for clarity.

In order to compare the results with and without the wetting layer, we performed calculations following our approach from Ref.^62^, where typical nanostructures located on a wetting layer were studied. In this procedure, shape anisotropy is applied by elongating the system along the [$1{\bar{1}}0$] axis and simultaneously narrowing it in the perpendicular [110] direction, with the quantum dash volume (base field) kept constant (Fig. 1). in such a manner a cylindrical disk-shape quantum dot undergoes a transition to an elongated quantum dash with an elliptic base. The anisotropy^16,62,64^ parameter *t* defines the elongation of the nanonstructure, and defines its length $X=r\,\left( 1+t\right)$ and width $Y=r/\left( 1+t\right)$, as depicted in Fig. 1. The details regarding quantum-dash dimensions, and other computational matters are discussed later in the text.

### Single particle states

**Figure 2:**
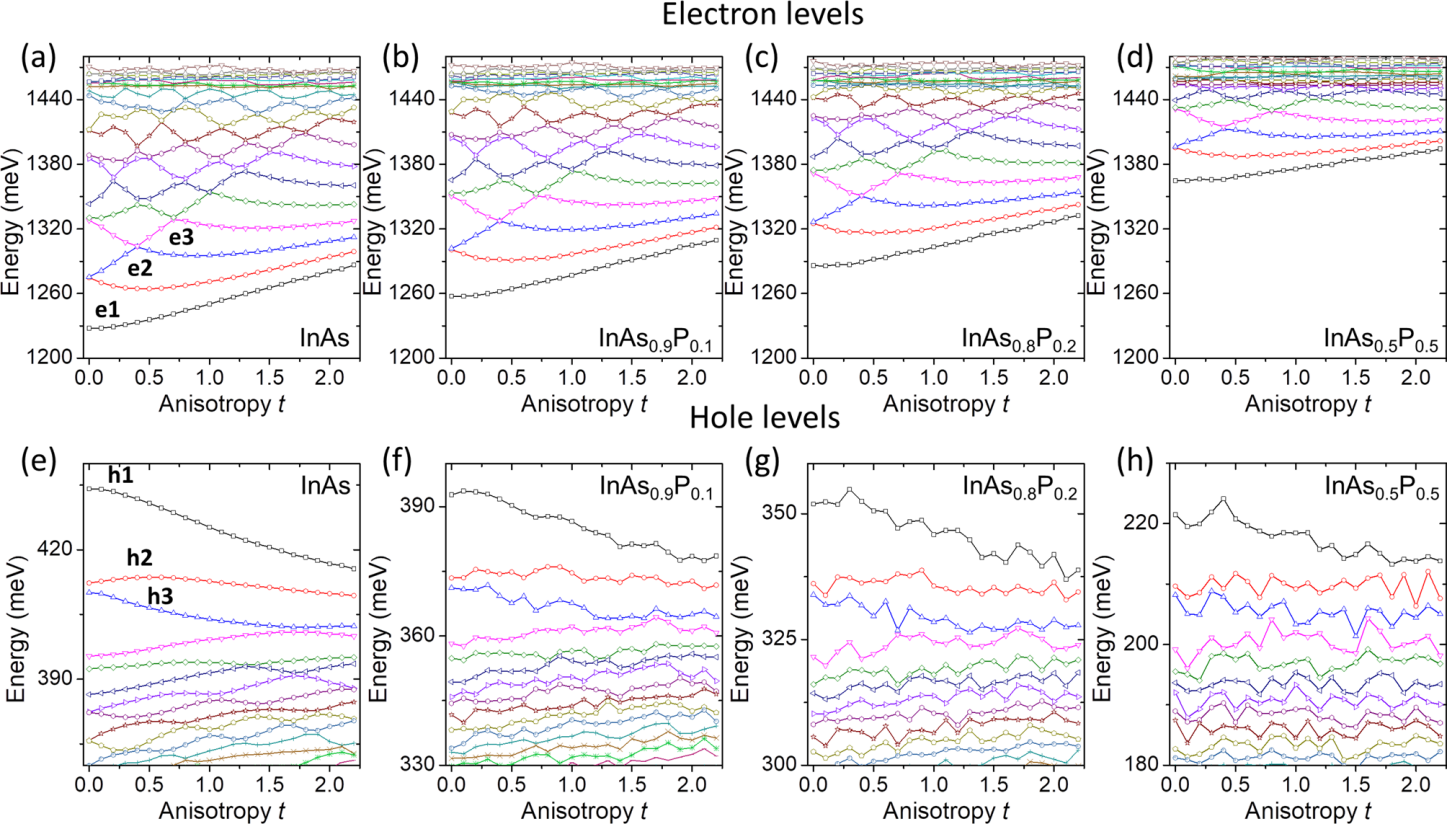
Single particle electron (**a**–**d**) and hole (**e**–**h**) levels as a function of elongation *t* and phosphorous content *P*. Please note variable energy scales, as well as reversed ordering of hole levels. Lines connect and order the states energetically.

Let us start by inspecting single-particle electron and hole levels as a function of both elongation and alloying, i.e. phosphorous content, as shown in Fig. 2. Electron levels demonstrate a transition from quasi-two-dimensional^2^ to quasi-one-dimensional one as a function of elongation, which is similar to the results obtained for a system with a wetting later^63^, depicting the change from a quantum-dot to a quantum-dash system. With the increasing deformation the energy of electron levels increases despite keeping the quantum dash base field fix. This is due to stronger confinement in the [110], which is not compensated by the increase of nanostructure length (along [$1{\bar{1}}0$]). This may be understood in a simple box or anisotropic harmonic confinement model, where confinement in each of the axes contributes as $l^{-2}$ (*l* is the confinement length) to the ground-state energy. Thus, fixed $X\,Y$ does not imply a constant value of $X^{-2}+Y^{-2}$, which is an increasing function of *t*. Notably, with an increasing alloying, i.e. amount of phosphorous (high energy-gap material) the confining potential becomes effectively shallower. Thus, electron states are shifting up in energy with increasing P content. Apart from that, the general structure of electron states appears to be weakly affected by alloying, with small fluctuations due to alloy randomness. Decrease of confinement leads, however, to a decreased number of confined states, which changes from $\sim 10$ for the unalloyed case to $\sim 5$ for $P=0.5$, with the onset of quasi-continuum states at about 1450 meV in all considered cases.

In contrary, hole levels (Fig. 2e–h) are strongly affected by alloying what appears to be a general feature of InAs/InP nanostructures^62,69,74^ likely due to smaller inter-level spacing of hole states, as compared to electrons. As a result, energies of excited hole states for alloyed systems (in particular for $P=0.2$ and $P=0.5$) are dominated by alloy randomness, with a weak dependence on anisotropy.

### Exciton-energy spectra

**Figure 3:**
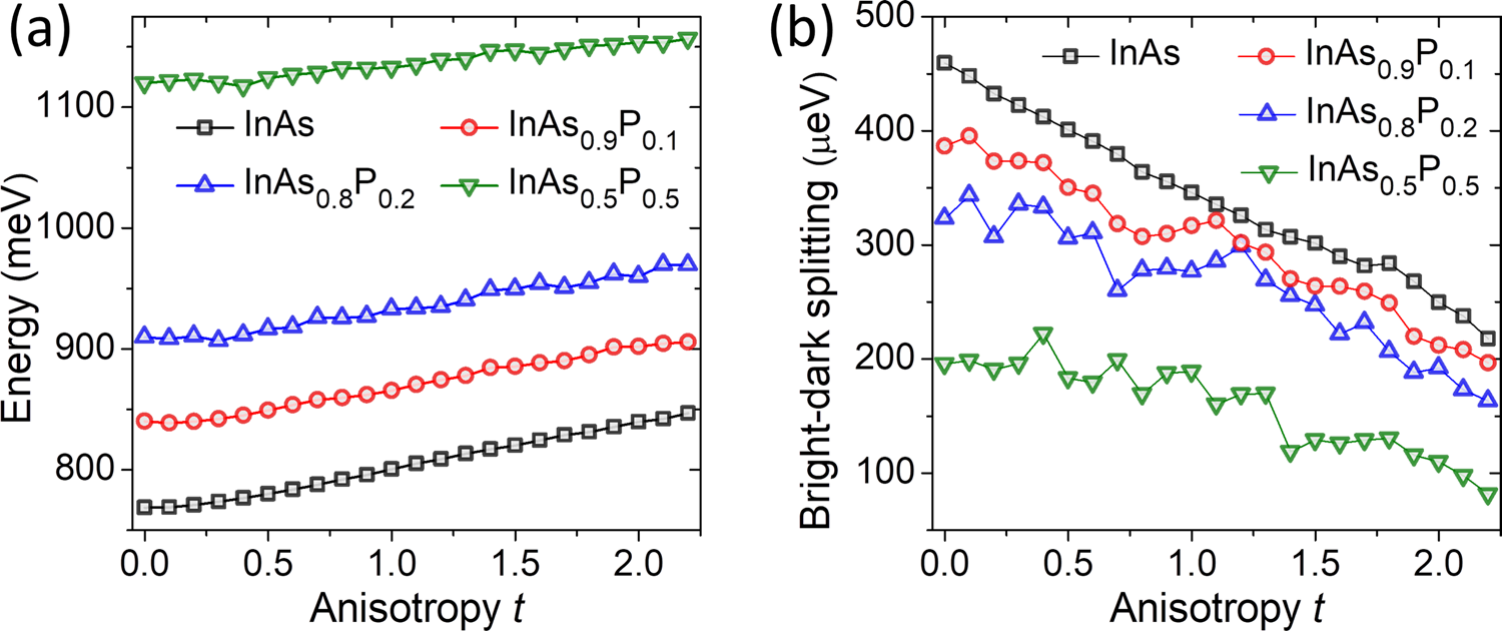
(**a**) Exciton ground-state energy and (**b**) bright-dark exciton state splitting as a function of quantum dash elongation *t* and phosphorous content *P*.

**Figure 4:**
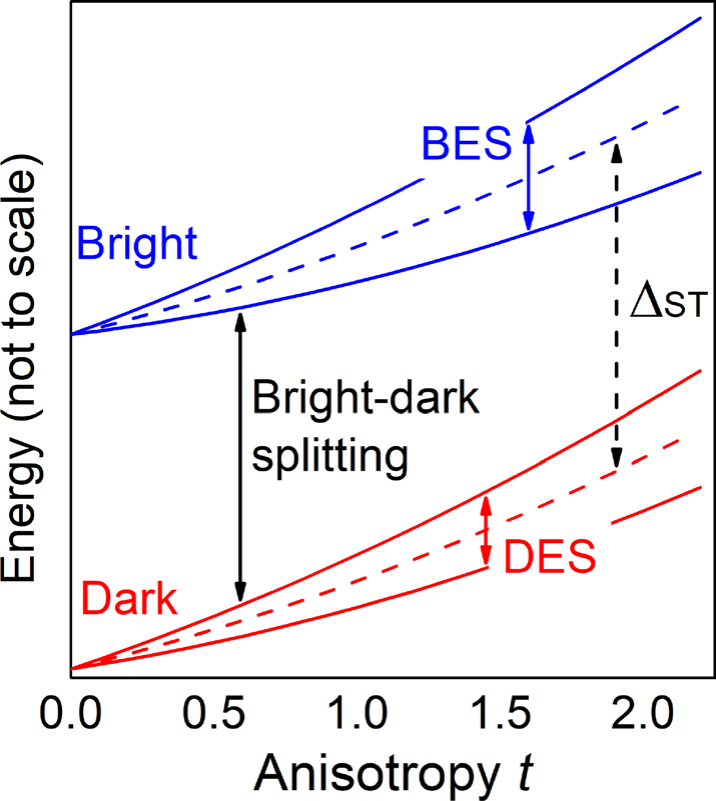
Schematics of excitonic energy levels ordering illustrating the bright exciton splitting (BES), dark exciton splitting (DES), bright-dark exciton splitting, and $\Delta _{\mathrm {ST}}$ used for simple modeling as described in the text. The bright-dark splitting is affected by splittings within bright and dark exciton manifolds, whereas $\Delta _{\mathrm {ST}}$ is not.

Figure 3a shows the ground-state energy of the exciton confined in an elongated quantum dash as a function of anisotropy *t* and alloying *P*. Energy of exciton levels increases with both deformation and P content, consistently with the behavior of single particle states (in particular electron) forming the exciton. As a result, only small fluctuations due to alloy randomness are present in the exciton ground-state evolution for large values of *P*. However, this picture gets more complicated when the details of excitonic spectra are studied, starting with the energy difference (Fig. 3b) between manifolds of bright and dark excitonic species (related to isotropic electron-hole exchange interaction^15^, schematically illustrated in Fig. 4). The bright-dark exciton splitting (Fig. 3b) is approximately reduced by half by alloying for $P=0.5$, consistent with an intuitive understanding in which an $\hbox {InAs}_{0.5}\hbox {P}_{0.5}$ nanostructure has the depth of confining potential twice lower than a pure InAs system. Moreover, this splitting is affected by alloying with notable fluctuations on top of the dominant deformation-related trend. Furthermore, in all considered cases the dark-bright splitting is significantly reduced by elongation. In particular, the magnitude of this splitting for maximally elongated system is approximately half of that for the symmetric case.

#### BES: no alloying

**Figure 5:**
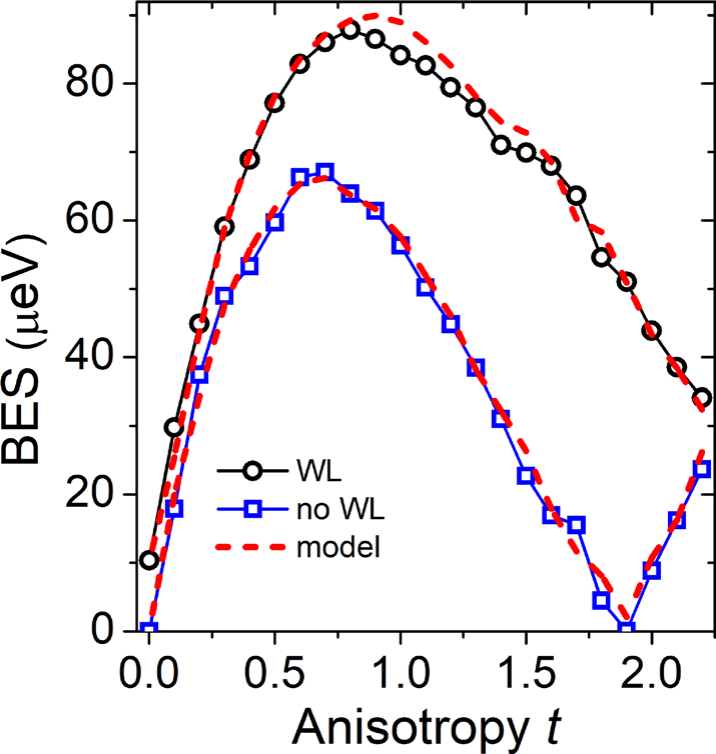
Comparison of the bright exciton splitting as a function of shape elongation for the case with (black circles) and without the wetting layer (blue equares).

According to the definition, the bright-dark splitting is to a certain degree affected by splittings within bright and dark excitonic doublets (Fig. 4). Moreover, the BES is the experimentally relevant quantity, thus let us now focus on it, as shown in Fig. 5. For sake of comparison, the BES is studied here for the cases with and without the wetting layer, whereas the effects of alloying will be studied further.

For a symmetric ($t=0$) and unalloyed nanostructure without the wetting layer, there is no fine structure splitting due to high $D_{2d}$ symmetry^52^. With elongation the symmetry is reduced to $C_{2v}$ leading to a non-zero BES, that is increasing with elongation and reaches the maximum of $\sim 67\,{\upmu }\hbox {eV}$ for $t=0.7$. Then it is quenched and decreases with further elongation down to a small value of $\sim 0.05\,{\upmu }\hbox {eV}$ for $t=1.9$. For even higher shape deformations, the polarization of excitonic lines (studied further in the text) is actually reversed, which indicates that BES is not simply reduced, but crossed zero, while the ordering of bright excitonic lines is reversed.

For the cases with the wetting layer, the symmetry is $C_{2v}$ even for $t=0$, which results in non-zero BES of $\sim 10\,{\upmu }\hbox {eV}$. This $\sim 10\,{\upmu }\hbox {eV}$ difference between both cases holds up to $t\simeq 0.25$, whereas for higher deformations the BES is significantly larger in nanostructures grown on the wetting layer. In both considered cases the BES has a maximum at $t\simeq 0.7$, and then it is quenched. For a nanostructure with a wetting later one could extrapolate the results of our calculation and expect vanishing fine structure splitting for an extraordinarily large anisotropy of $t\simeq 3$ (aspect ratio of 16 to 1) corresponding to a technologically challenging growth of nanostructures with lateral widths of only 5 nm and less. This in different than in the cases without the wetting layer, where fine structure splitting vanishes already for $t=1.9$, and thus for aspect ratio of $\sim 8$ and lateral thickness of over 7 nm, thus corresponding to more realistically attainable dimensions.

While our results are obtained in a multi-band atomistic calculation accounting for configuration mixing effects, the key effect of vanishing fine structure splitting is also present in an approximation, where only the lowest electron and hole states, i.e. *s* shells, are accounted for (see the “Appendix”). Thus our results could in principle be analyzed in terms of a simple theoretical model. As proposed in Ref.^75^, the bright exciton states could be given as:

1 $$\begin{aligned} \left| \pm 1\right\rangle =\sqrt{1-\beta ^2}\left| \pm \frac{3}{2},\mp \frac{1}{2}\right\rangle +\beta \left| \mp \frac{1}{2},\mp \frac{1}{2}\right\rangle , \end{aligned}$$

where $\left| \pm \frac{3}{2},\mp \frac{1}{2}\right\rangle$ are the heavy-hole exciton states with a $\beta$ add-mixture of $\left| \mp \frac{1}{2},\mp \frac{1}{2}\right\rangle$ light-hole excitons. In a simple two-by-two effective Hamiltonian model^75^, expressed in a basis of Eq. 1, the magnitude of BES is given as

2 $$\begin{aligned} BES=\left| \Delta _{\mathrm {hh}}\left( 1-\beta ^2\right) +\Delta _{\mathrm {lh}}\beta ^2+ \frac{4}{\sqrt{3}}\beta \sqrt{1-\beta ^2}\Delta _{\mathrm {ST}}\right| , \end{aligned}$$

where $\Delta _{\mathrm {ST}}$ is the non-anisotropic electron-hole exchange energy related to the dark-bright exciton splitting, as shown schematically in Fig. 4, and mediating the relation between isotropic and non-isotropic fine structure splitting, whereas $\Delta _{\mathrm {hh}}$ and $\Delta _{\mathrm {lh}}$ are contributions related to heavy- and light-hole states, correspondingly, and not having a direct relation to $\Delta _{\mathrm {ST}}$. The mixing parameter $\beta$ can obtained from optical spectra of bright excitonic states (discussed further in the text), namely the polarization anisotropy^75–77^

3 $$\begin{aligned} C=\frac{I_{max}-I_{min}}{I_{max}+I_{min}}=\frac{2\beta \sqrt{3\left( 1-\beta ^2\right) }}{3-2\beta ^2} \end{aligned}$$

For an unalloyed ideally cylindrical ($t=0$) case without the wetting layer (disk-shaped, pure InAs quantum dot) the resulting symmetry is $D_{2d}$, and the fine structure splitting vanishes^52,53^, as both $\beta =0$ and $\Delta _{\mathrm {hh}}=\Delta _{\mathrm {lh}}=0$. For elongated systems ($\beta \ne 0$) the symmetry is reduced to $C_{2v}$, yet one could use an approximation in which still $\Delta _{\mathrm {hh}}=\Delta _{\mathrm {lh}}=0$, and thus $BES \approx \left| \frac{4}{\sqrt{3}}\beta \sqrt{1-\beta ^2}\Delta _{\mathrm {ST}}\right|$. This approach partially explains the reduction of the bright exciton splitting with elongation in terms of a competition between increasing $\beta \sqrt{1-\beta ^2}$ contribution due to light-hole content and $\Delta _{\mathrm {ST}}$ following the bright-dark splitting (Fig. 3a), and thus decreasing^63^ with elongation. However, such approximation does not reproduce the case with a vanishing (or reversed) fine structure, and moreover such model systematically and significantly overestimates the magnitude of the BES as compared to atomistic calculations^63^. To overcome both issues, we propose to use a formula with $\Delta _{\mathrm {lh}}\ne 0$,

4 $$\begin{aligned} BES=\left| \Delta _{\mathrm {lh}}\beta ^2+ \alpha \frac{4}{\sqrt{3}}\beta \sqrt{1-\beta ^2}\Delta _{\mathrm {ST}}\right| , \end{aligned}$$

in which the last term has been scaled down (corrected) by parameter $\alpha$ in order to overcome systematic error of an simple model, which is mostly related to fact the atomistic calculation are performed in a larger excitonic basis, thus accounting for effect of configuration mixing, which are absent in a 2-by-2 Hamiltonian treatment of Ref.^75^. Results of this phenomenological approach are shown in Fig. 5 with dashed red lines presenting a very good fit to the atomistic results. Here, we used effective parameters $\Delta _{\mathrm {lh}}=-340\,{\upmu }\hbox {eV}$ and $\alpha =0.37$. (We note here that we could also obtain a fit of similar quality by using even simpler model, i.e., without introducing using $\alpha$ parameter, but by treating $\Delta _{\mathrm {ST}}$ as constant (not depending on elongation) equal to 185 meV, and by setting $\Delta _{\mathrm {lh}}=-573\,{\upmu }\hbox {eV}$.) Furthermore, the same approach works well also for the case with the wetting layer (again plotted with dashed red line in Fig. 5), with $\Delta _{\mathrm {lh}}=-270\,{\upmu }\hbox {eV}$, and $\alpha =0.435$. Additionally, due to the $C_{2v}$ symmetry of a nanostructure located on a wetting layer, even for $t=0$ one must add a heavy-hole term (i.e. $\Delta _{\mathrm {hh}}\left( 1-\beta ^2\right)$) responsible for the non-zero fine structure spitting even in a fully cylindrical case. In our case $\Delta _{\mathrm {hh}}=7.1\,{\upmu }\hbox {eV}$, which is significantly smaller than $\Delta _{\mathrm {lh}}$, and actually plays a role only for very weakly elongated cases.

Thus, the BES in highly elongated systems, in particular for the case without the wetting layer, appears to be governed by a competition of two light-hole terms: the quasi-linear term $\beta \sqrt{1-\beta ^2}\Delta _{\mathrm {ST}}$ dominant for moderate elongations, and the quadratic term $\Delta _{\mathrm {lh}}\beta ^2$ that prevails for higher anisotropy, effectively allowing for the swapping of excitonic lines, and the vanishing BES occurring in the process. For nanostructures grown on the wetting layer it is possible for single particle states to leak into it^63,78^, which effectively partially compensates the anisotropy due to elongation. This leakage is, however, not possible for systems without the wetting layer, presumably allowing for a pronounced reduction of the BES for considerably smaller and more realistic values of *t*.

Results presented here suggest that it is in principle possible to obtain very low bright exciton splitting for highly shape-anisotropic nanostructures, grown without or electronically decoupled from the wetting layer. Quantum dashes with strongly reduced bright exciton splitting should in principle be very good candidates for further tuning of this splitting to zero by application of external fields^18,55–61^. This, combined with recent experiments on the control of degree of polarization^46^ via photonic mesas, should open paths towards efficient generation of entangled photon pairs using quantum dashes. The reduction of the bright exciton splitting was shown here for a relatively narrow quantum dash with $t=1.9$ corresponding to the width of 7 nm. Determining the degree of agreement between the theory and the experiment by accounting for alloying effects^79,80^ is a promising path of research, and hence effects of alloying are studied in the following section.

#### BES: alloying

**Figure 6:**
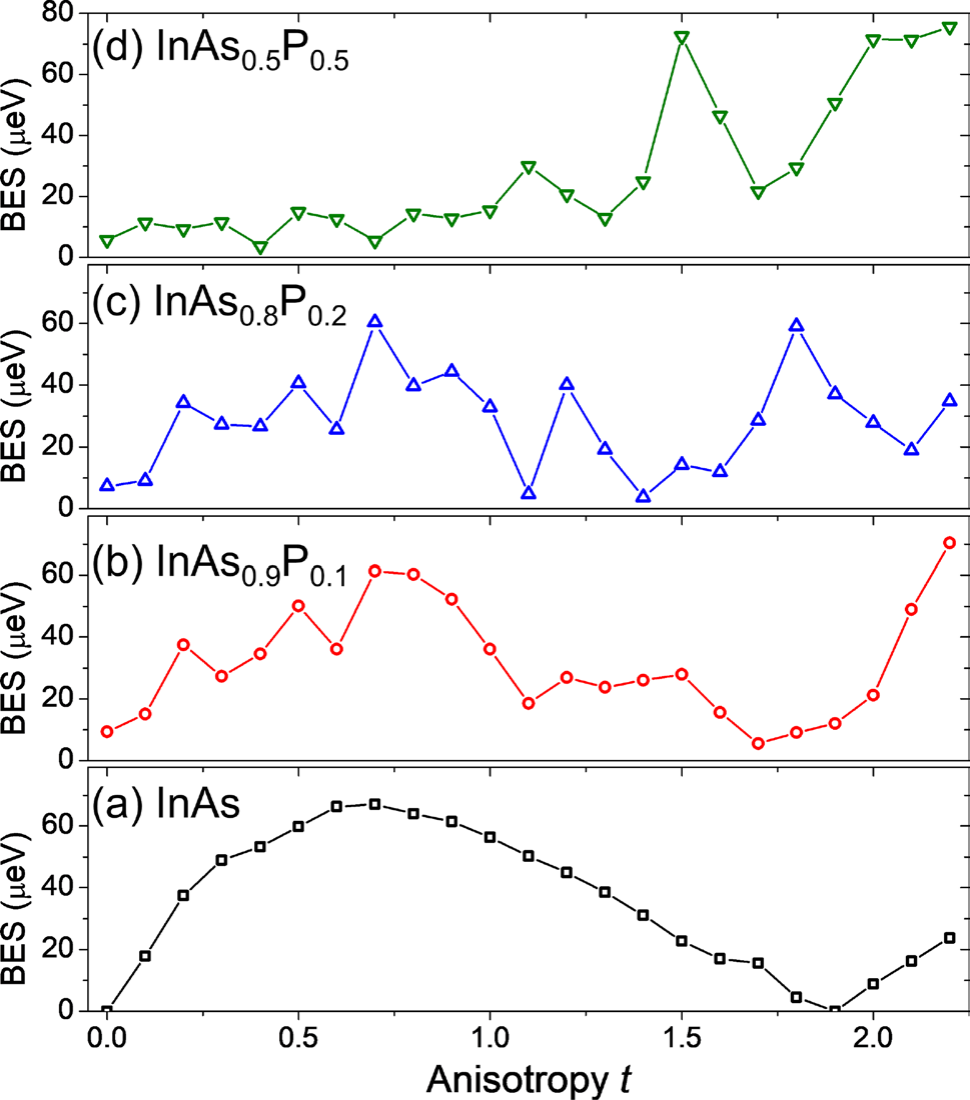
Bright exciton splitting as a function of anisotropy *t* for various P contents. See the text for details.

Alloy randomness has a pronounced effect on the BES, as shown in Fig. 6. The BES is studied here as a function of elongation and P content. With low alloying $P=0.1$, the general trend on shape-deformation is still present, for $P=0.2$ both alloy randomness and the reduction of BES for larger *t* values seem to be comparable, whereas for $P=0.5$ alloy randomness appears to dominate and smears the reduction of the BES with elongation. This “smearing” due to alloying is actually even more pronounced in a simplified theoretical treatment, where one neglects configuration mixing with higher shells (see the “Appendix”), and where the BES evolution with anisotropy for the $P=0.5$ case is quasi-linearly increasing with deformation, rather than being reduced for large *t* values.

Therefore, in the alloyed case the BES spectra could be viewed as an interplay of three “terms”. Two of these (related to light-hole contribution) apparently compete against each other as has been discussed for the non-alloyed case, and the third “contribution” being the alloying. For $P=0.5$ alloying, and for $t<1$, alloy randomness is effectively reducing the anisotropy, and thus apparently dumping the BES increase with deformation, apart from adding notable random oscillations. Yet, for even higher elongations one of shape elongation related contributions tends to dominate, and it prevails over alloying as well, leading to high BES values for $t>2$, what is mostly mediated by presence of higher shells (see the “Appendix”). We note however here, that more extensive studies on role of alloying (especially for large *t* values) need the be performed, e.g. by considering more than a single sample corresponding to the same average composition^68,69^. Due to large number of atoms, and related computational complexity of the configuration interaction these are ongoing studies, and their results will be presented elsewhere.

**Figure 7:**
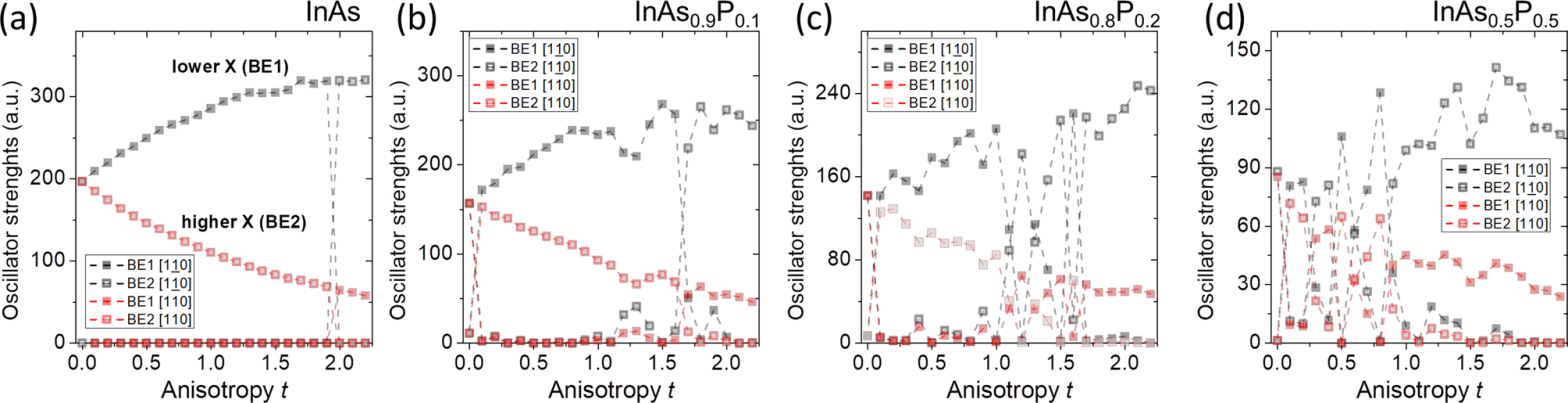
Bright exciton optical spectra as a function of anisotropy *t* for various P contents. See the text for details.

Pronounced role of alloying is also present in the bright exciton optical spectra, as shown in Fig. 7. For unalloyed and fully cylindrical case (Fig. 7a), both optically active excitonic lines have the same height, thus there is no polarization anisotropy (defined earlier in Eq. 3 as *C*). With the increasing shape elongation the lower excitonic line (BE1) is polarized along [$1{\bar{1}}0$] direction, and gets higher oscillator strength, whereas the energetically higher excitonic line (BE2) has orthogonal polarization ([110]) with lower strength. This difference in oscillator strengths leads to polarization anisotropy that grows with shape anisotropy, and we had used that earlier to retrieve $\beta$ parameter. At $t=1.9$ excitonic lines reverse their energetic order, and now BE2 line is polarized along [$1{\bar{1}}0$], whereas BE1 along [110]. Notably, despite the $C_{2v}$ symmetry, both bright excitonic lines can cross (rather than anti-cross), since for unalloyed quantum dots bright excitonic states belong to different irreducible representations^81^. Hence, based on group-theoretical reasoning and contrary to simplified arguments shape deformation can in fact lead to a vanishing fine structure splitting.

The pictures gets more complicated for alloyed systems with polarization axes getting randomized^69,82^. However, for larger *t* values the bright exciton polarizations still maintain their dominant components in the [$1{\bar{1}}0$]/[110] basis, therefore we present these components on Fig. 7b–d. Figure 7b shows (for $P=0.1$) that the dip in the BES for $t=1.7$ is in fact assisted by the reversal of polarization even in this alloyed case. Similar effect occurs as well for $P=0.2$ (Fig. 7c; e.g. $t=1.1$) confirming that in principle the reduction of the BES with anisotropy may occur even in realistic cases with random alignment of atoms, provided the alloying level is limited. Interestingly, for alloyed cases there is also a reversal of polarizations for bright excitonic lines happening between $t=0$ and $t=0.1$. This is due to the fact that in alloyed cases there is a non-zero (7 to $10\,{\upmu }\hbox {eV}$) BES already for $t=0$ due to alloying^69^, and in cylindrical alloyed cases the lower excitonic line is predominantly polarized along the [110] direction, thus reversely to elongated cases. Therefore, small elongation of $t=0.1$ reverses polarization of excitonic lines that is imposed by alloying in cylindrical nanostructures. This effects is somewhat similar to reversal of polarization in $C_{2v}$ systems located on the wetting layer studied in our earlier work^62^. However, it should be reiterated that with alloying and low $C_1$ symmetry, polarization axes are matching crystal axes only approximately with unavoidable randomization^82^. For $P=0.2$ this randomization is more notable, yet one can still observe that minima in the BES (Fig. 6d) are assisted by corresponding changes of polarization properties (Fig. 7d). Finally, for $P=0.2$ the randomization is quite strong for $t<1$, and generally the picture is complicated for smaller elongations, yet interestingly for larger *t* excitonic lines get very similar polarization properties as those observed for highly elongated cases with $P=0$, 0.1 and 0.2. Thus, shape anisotropy appears to enforce polarization properties for highly elongated, even though alloyed systems.

### Dark exciton

**Figure 8:**
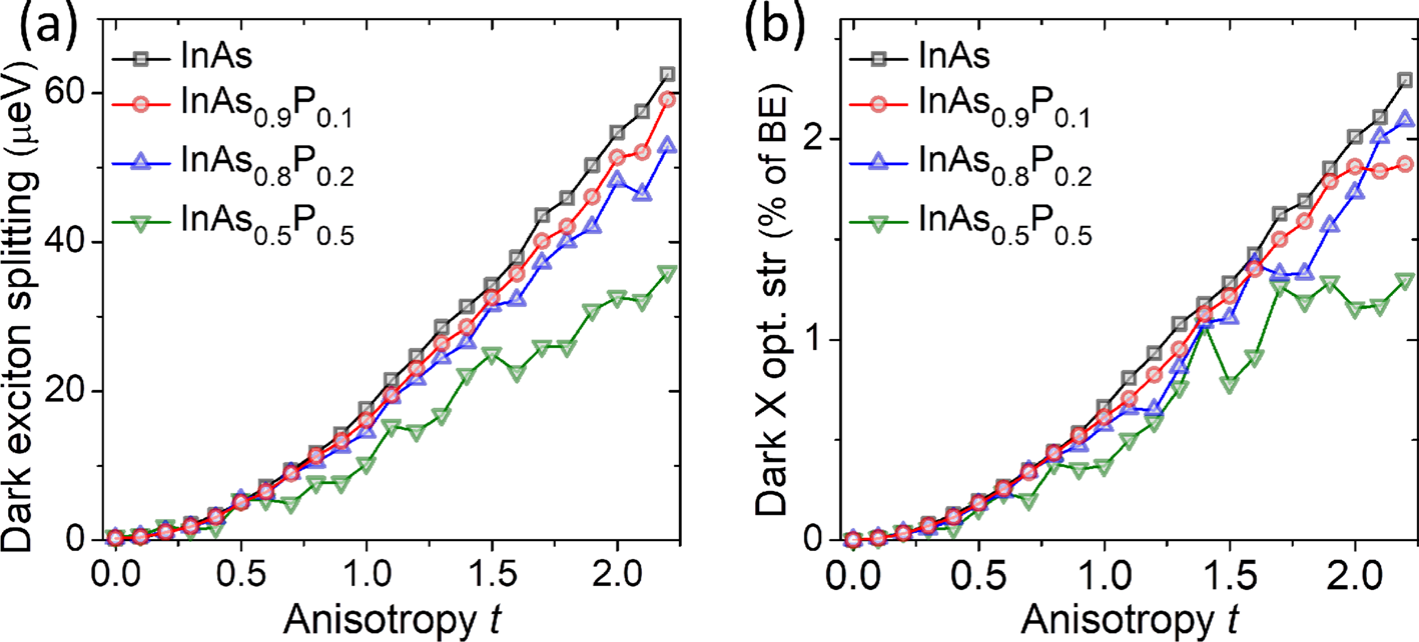
Dark exciton energy splitting (**a**) and optical activity (**b**) as a function of anisotropy *t* and alloying for vrious P contents. Optical activity is given as oscillator strength reslative to (percentages) the bright-exciton oscillator strength for non-elongated cases. See the text for more details.

Dark excitons are nowadays considered as important platform for various quantum-dot applications^70–72,83–88^. In Fig. 8, we show dark exciton splitting (DES) and its optical activity, both (quasi-)monotonically increasing with deformation, thus very similar to systems grown on the wetting layer. For the dark exciton the effect of alloying is substantial only for $P=0.5$, and only for larger deformation, where it apparently reduces the DES. In alloyed cases there are also some fluctuation due to alloy randomness, yet contrary to the BES, the overall trends of the DES and increase of the optical activity of dark exciton with elongation are to a large degree immune to alloy randomness.

In a simple model^63,75^ treatment the DES should increase proportionally to the light-hole content ($\beta$), and thus monotonically increase with *t*. Therefore, for non-alloyed system, there is no competing term that could reduce the DES, as it happened for the BES, and thus the role of alloying appears to be less pronounced on the DES. The DES is also largely affected by the presence of higher-shells (see the “Appendix”) which is not accounted in the simple modeling. We found that configuration mixing with *p*- and *d*-shells is responsible for additional the increase of the DES for $t>1$ (addition of the *f*-shell apparently does not play a significant role; see the “Appendix”). In effect, the DES evolution is non-linear with *t*, growing rather weakly for small elongations, and more rapidly for $t>1$ (Fig. 8a). This effect is somewhat flatted by alloying, yet it is present even in the highly-alloyed case.

The different behavior of bright and dark complexes with respect to anisotropy is consistent with the lack of coupling between these excitonic species, as here we consider structures with high shape symmetry^72^, and thus the dark and bright excitonic manifolds are independent from each other. Notably the optical activity of the dark exciton reaches substantial 1 to 2% fraction of the bright exciton optical activity, for highest considered elongations, even in alloyed cases. Consistently with group-theoretical considerations^54,73^, for non-alloyed $C_{2v}$ systems one of dark excitonic lines (DE1, energetically lower) is strictly “dark” (optically inactive), whereas the energetically higher dark excitonic line (DE2) has non-zero optical activity, yet it is strictly *z*-polarized (with the exception of $t=0$ and $D_{2d}$ symmetry, where both dark excitons are strictly optically inactive).

For alloyed systems the optical activity of the dark exciton is still strongly dominated by the emission from energetically higher dark state (DE2) and is predominately *z*-polarized with only negligible emission with other polarizations, and virtually negligible emission from energetically lower (DE1) dark exciton state.

## Discussion

We considered here quantum dash systems studied as a function of lateral aspect ratio and chemical composition. Nanostructures studied in this paper are assumed to be grown without the wetting layer, and to be disk-shaped in the growth direction. Lack of the wetting layer and a flat geometry leads to high overall symmetry, and thus contributions discussed here that lower the symmetry are related to (1) lateral deformation along one of the crystal axes, and (2) alloy randomness for chemical compositions different from pure InAs.

Our results indicate that shape anisotropy and disorder due to alloy randomness appear to compete regarding their impact on the fine structure splitting, whereas interestingly the shape-anisotropy may in fact lead to its vanishing. This surprising result can be understood in terms of a phenomenological model with two counteracting light-hole contributions of opposite signs. The magnitude of one of these contributions grows quadratically with shape elongation, and thus it dominates for larger aspect ratios. At a certain elongation these terms mutually cancel, which leads to a vanishing fine structure splitting. The reduction of the BES is assisted by the reversal of bright excitonic lines polarizations, yet large polarization anisotropy remains present even when BES is reduced. Generally, the polarization anisotropy grows with deformation similarly as in the case of more conventional quantum dashes located on a wetting layer.

Alloying counteracts strong spatial anisotropy and thus it appears to blur well defined BES trends. However, for small level of P contents, the bright exciton energy and optical spectra evolution with *t* qualitatively resembles that for pure InAs quantum dash. Thus, reduction of BES in elongated systems is still possible with alloyed system, provided that the level of material intermixing is sufficiently small. Alloyed quantum dashes maintain strong polarization anisotropy, with polarization axes randomized, yet still predominately related to the elongation/crystal axes. Finally, the dark exciton spectra appear to be largely immune to alloying, and being dominated by shape anisotropy.

To summarize, our theoretical results suggest that elongated nanostructures, yet without (or decoupled from) the wetting layer, with flat geometry and low alloying are good candidates for further research aiming for the BES reduction in InAs/InP semiconductor nanostructures.

## Methods

The diameter of a quantum dash in a fully cylindrical (i.e. quantum dot) case is 20.6 nm, i.e., the radius is $r=10.3\,\hbox {nm}$. The height of all deformed nanostructures is kept fixed at 3 nm. We consider *t* to vary from 0 to 2.2 (see Fig. 1), so the aspect ratio is $X/Y=(1+t)^2$ and reaches over 10 for the highest deformation considered. In order to account for the effect of alloying, apart from pure InAs system, we repeated the same calculation, yet for there different phosphorous contents: 10, 20 and 50%, i.e., corresponding to $\hbox {InAs}_{0.9}\hbox {P}_{0.1}$, $\hbox {InAs}_{0.8}\hbox {P}_{0.2}$, and $\hbox {InAs}_{0.5}\hbox {P}_{0.5}$. The choice of alloying levels (and choice of one random sample per each *t* and *P* value) was limited by the significant computational complexity of each calculation. Since *t* was varied from 0 to 2.2 with a step size of 0.1, the calculations were performed for a total of 92 different systems. For a non-alloyed case, the results without the wetting layer were compared with those accounting for the wetting layer as published in Ref.^63^. Only two additional data points ($t=2.1$ and $t=2.2$) were calculated for this case.

Our calculation starts with finding atomic positions that minimize the total elastic energy of the lattice, by using the valence force field method of Keating^89–93^. Next, from atomic positions the piezoelectric potential is calculated^94–98^, with piezoelectricity model and coefficients taken from Ref.^96^. Then, the single particle spectra of electrons and holes are obtained with the empirical tight-binding method accounting for up to *d*-orbitals and spin-orbit interaction^78,78,91,99,99,100,100–103^.

Finally, the excitonic spectra are calculated with the configuration interaction method^2,104–108^. Configuration interaction calculations are performed in a computationally challenging basis^63^ involving 20 (with spin) lowest-energy electron and hole states (up to the *f* shell of a single cylindrical quantum dot) and leading to the total 400 excitonic configuration, wheres the largest computational cost is related to calculation of 160,000 electron-hole Coulomb integrals^105,106^ over a computational box containing over $1.3\times 10^6$ atoms. Results obtained in a smaller basis of lowest 4 excitonic configurations are shown in the “Appendix” for comparison.

## Figures and Tables

**Figure 9:**
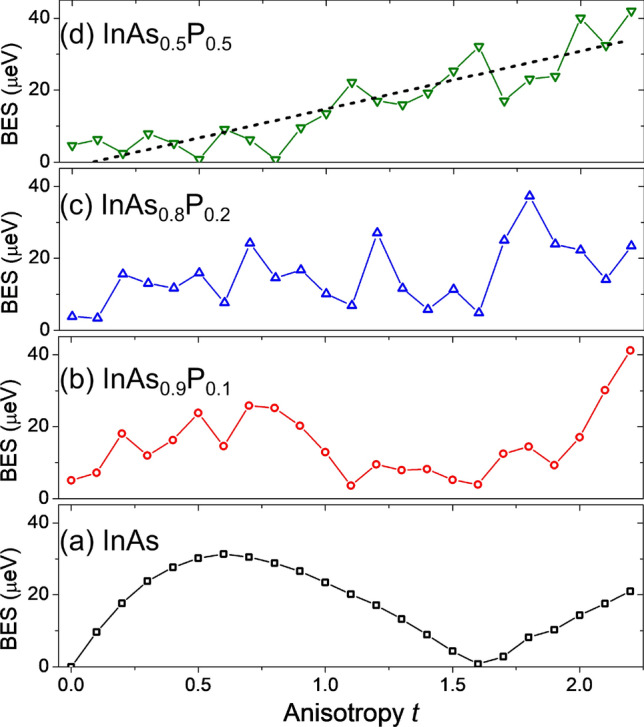
Bright exciton splitting as a function of anisotropy *t* for various P contents, for a calculation that neglects configuration mixing with higher states (*p*, *d*, and *f*-shells). See the text for details.

**Figure 10:**
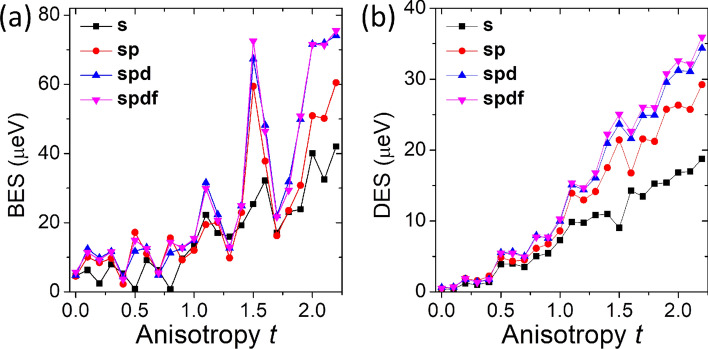
Bright (**a**) and dark (**b**) exciton splitting calculated for $\hbox {InAs}_{0.5}\hbox {P}_{0.5}$ nanostructure by assuming different number of states (*s*, *p*, *d*, and *f*-shells) included in configuration interaction calculation. See the text for details.

## Appendix 1: BES: s-shell only

The BES in elongated systems is strongly affected by contributions from higher-energy shells via configuration mixing^63^. In order to study this effect for systems without the wetting layer, we show in Fig. 9 the results of the BES calculation similar to those studied earlier in Fig. 6, yet for a situation where configuration mixing with higher shells in not accounted for. Namely, Fig. 9 was obtained in a $4\times 4$ excitonic basis^15^ by including the (electron and hole) *s* shells only. Figure 9 shows that generally results in a limited configuration-interaction basis, especially for the unalloyed case, are qualitatively similar to those obtained by a more complete treatment. The reduction of the BES with *t* is thus not related to configuration mixing, but occurs already in a simplified treatment including the ground states only. However, mixing of configurations (in other words correction due to correlations) generally increases the BES by (approximately) factor two, with more quantitative differences occurring for alloyed nanostructures. In particular, for a strongly alloyed case of $P=0.5$, the BES trend is strongly “flattened” or “smeared out” by alloying, and to a large degree is quasi-linear with respect to shape anisotropy *t*, yet with some fluctuations. This trend is qualitatively very different from the unalloyed case, which has a strong dip and reversal of the BES for $t=1.6$. Such dependence of the BES that grows quasi-monotonically with anisotropy, with only some minor fluctuations due to disorder is consistent with an effective-mass treatment of the problem^64^. However, such intuitive approach is based on virtual crystal approximation for alloying, as well as lack of mixing with higher shells. Accounting for both effect leads to a much complicated results as discussed in the current work.

Moreover, without alloying the reversal of bright excitonic lines occurs already for $t=1.6$, as compared to $t=1.9$ when accounting for higher shells. Thus, limiting number of higher-energy states should in principle reduce the BES via reduction of correlations, and directly due to lack of higher-energy states involved in the mixing. This could be experimentally achieved, e.g., with smaller quantum dot/dash heights. From a theoretical point of view this is an interesting direction of research that could be continued by considering nanostructures of different heights or shapes. Such approach combined with decoupling of the wetting layer from quantum dash spectra could be beneficial for tailoring of low BES system, similar to tailoring number of charged excitons in systems without the wetting layer.^67^

## Appendix 2: BES and DES: higher shells

To further study the role of the configuration interaction Fig. 10 shows results of the BES and DES calculations for $\hbox {InAs}_{0.5}\hbox {P}_{0.5}$ nanostructure assuming a different number of single particle states (*s*, *p*, *d*, and *f*-shells) used in the calculation, and leading to an increasing number of excitonic configurations from 4, 36, 144 to 400 respectively. Figure 10 shows that contributions from *p*-, and *d*-shells should to be accounted for aiming for more accurate results, whereas the *f*-shell apparently plays a lesser role, making it probably that going for even higher levels (”*g*-shell”) is likely unnecessary, and therefore suggesting that configuration interaction procedure is close to convergence, at least with respect to considered spectral quantities.

Finally, we note that for the DES, the configuration mixing (i.e. going being the *s*-shell) is particularly important $t>1$, whereas for the BES it apparently needs to included for in the entire range of elongations. We also note that accounting for *s*- and *p*-shells only gives a very good trade-off between accuracy and computational complexity, i.e. a relatively good “quantitative” description of the BES/DES evolution, that comes with less than 1% cost of Coulomb and exchange integrals calculations (since the number of integrals that needs to be calculated scales as fourth-power^105–107^ of single particle states included in the calculation).
